# Isolation of the *ZmERS4* Gene From Maize and Its Functional Analysis in Transgenic Plants

**DOI:** 10.3389/fmicb.2021.632908

**Published:** 2021-03-12

**Authors:** Tianlu Hang, Xuezhi Ling, Cheng He, Shanshan Xie, Haiyang Jiang, Ting Ding

**Affiliations:** ^1^National Engineering Laboratory of Crop Stress Resistance Breeding, School of Life Sciences, Anhui Agricultural University, Hefei, China; ^2^Anhui Province Key Laboratory of Integrated Pest Management on Crops, Key Laboratory of Biology and Sustainable Management of Plant Diseases and Pests of Anhui Higher Education Institutes, School of Plant Protection, Anhui Agricultural University, Hefei, China

**Keywords:** maize, *ZmERS4*, ethylene receptor, disease-resistant, salicylic acid

## Abstract

A gene encoding a protein similar to ethylene receptor was isolated from maize (*Zea mays* L.), which was named as *ZmERS4*.The gene was 1,905 bp in length with an open reading frame that encoded a protein consisting of 634 amino acids. The homologous analysis showed that *ZmERS4* shared high similarity with the ethylene receptor protein, OsERS1, from rice (*Oryza sativa* L.). *ZmERS4* grouped into the ETR1 subfamily of ethylene receptors based on its conserved domain and phylogenetic status. Tissue-specific and induced expression analyses indicated that *ZmERS4* was differentially expressed in maize tissues, predominantly in the stems and leaves, and was induced by salicylic acid (SA). Overexpression of *ZmERS4* in *Arabidopsis* improved resistance against the bacterial pathogen, *Pst*DC3000, by inducing the expression of SA signaling-related genes. Moreover, treatment with flg22 induced the expression of the defense-related gene, *PR1*, in maize protoplasts that transiently expressed *ZmERS4*. Furthermore, the ultra-high-performance liquid chromatography (UPLC) analysis showed that the SA contents in *ZmERS4*-overexpressing *Arabidopsis* lines were significantly higher than the control lines. Additionally, the improved resistance of *ZmERS4*-overexpressing *Arabidopsis* against *Pst*DC3000 was blocked after pretreatment with the SA biosynthetic inhibitor, ABT. Based on the collective findings, we hypothesize that *ZmERS4* plays an important role in disease resistance through SA-mediated signaling pathways.

## Introduction

Plants were attacked of various pathogenic microorganisms, including fungi, bacteria, viruses, and nematodes, in their natural environment. Maize (*Zea mays* L.) is one of the most important food crops produced in the world and is a staple food for ~50% of the global population (Ranum et al., 2014). To date, major maize diseases, including southern corn leaf blight, brown leaf spot, northern corn leaf blight, *Curvularia* leaf spot, and rough dwarf, have caused severe yield losses in maize production worldwide (Subash, 2015). To address this problem, improving host resistance against pathogens is the most economical and environmentally friendly approach. To breed resistant cultivars, efforts have aimed to introduce resistance (*R*) genes into susceptible cultivars. *R genes* are the key components of disease resistance against particular pathogens, which are often associated with hypersensitive responses (HR). According to “gene-to-gene” hypotheses, the *R*-genes of host plants interact with pathogen virulence effect factors to produce disease resistant phenotypes in the hosts (Sekhwal et al., 2015). However, owing to variations in pathogen virulence, resistant cultivars with *R* genes are effective for only a few years in agricultural production (Wang et al., 2013). Moreover, although several quantitative trait loci (QTLs; or quantitative genes) that confer disease resistance have been identified in maize, these sources have not been used effectively for maize improvement due to the complexity of controlling quantitative resistance. Therefore, breeding resistance cultivars with broad-spectrum and durable disease resistance is a top priority in maize improvement efforts around the world.

To date, >300 *R* genes have been identified and studied in *Arabidopsis*, tomato, rice, and other species (Kourelis and van der Hoorn, 2018). However, only a few genes have been identified as genetic resources for broad-spectrum disease resistance in maize. For example, *Hml* was first found in corn and enhanced plant resistance against northern leaf spot (Johal and Briggs, 1992). The maize resistance gene, *ZmRxo1*, which has a nucleotide-binding site-leucine-rich repeat structure, confers resistance to rice bacterial streak disease (Zhao et al., 2005). Li et al. identified an F-box protein (ZmFBL41) that confers maize resistance to banded leaf and sheath blight through a genome-wide association study, and *ZmFBL41*-overexpressing rice exhibited increased susceptibility to *Rhizoctonia solani*. Two amino acid substitutions in this allele prevent its interaction with ZmCAD, which encodes the final enzyme in the monolignol biosynthetic pathway and results in the inhibition of ZmCAD degradation and, consequently, the accumulation of lignin and restriction of lesion expansion (Li et al., 2019). Thus, genetic improvement and disease resistance cultivar breeding are current viable alternative strategies for crops suffering from biotic stress.

Ethylene is a gaseous hormone that regulates various processes during plant development including seed germination, root hair formation, leaf and petal abscission, fruit ripening, and senescence (Abeles et al., 1992). Little is known about the process of ethylene perception prior to the isolation of ethylene receptor genes. Ethylene receptors are homologous to bacterial two-component histidine kinase receptors (Bleecker, 1999) associated with the endoplasmic reticulum (ER) membrane (Chen et al., 2002, 2007; Ma et al., 2006) and have been confirmed to function as negative regulators of ethylene responses, which are inactivated by ethylene binding (Hua and Meyerowitz, 1998). Ethylene receptor genes have been identified in many plant species and classified into two subfamilies (Zhou et al., 1996; Hua and Meyerowitz, 1998). Subfamily I contain three transmembrane domains in the amino-terminal region and a well-conserved histidine kinase domain in the carboxy-terminal region. Subfamily II has four membrane-spanning domains and a degenerate histidine kinase domain that lacks one or more of the conserved amino acids, which are believed to be necessary for catalytic activities. The expression patterns of ethylene receptor genes have been examined and were found to be generally regulated according to tissue, developmental stage, and environmental stimuli (Hua and Meyerowitz, 1998; Chen et al., 2005).

In our previous work, the endophytic strain, *Bacillus subtilis* DZSY21, was isolated from *Eucommia ulmoides* and effectively colonized maize leaves and enhanced disease resistance (Ding et al., 2017). Key disease-resistance genes in the interaction system between maize and endophytic DZSY21 were identified by whole genome bisulfite and transcriptome sequencing (Zhou et al., 2019) and *ZmERS4* gene was one of them. In this study, the maize gene, *ZmERS4*, is isolated and characterized, and its expression patterns during development and under hormone treatments are investigated. The role of *ZmERS4* in conferring plant disease resistance is clarified, thus laying a foundation for cultivating resistant maize varieties.

## Materials and Methods

### Bioinformatics Analysis of *ZmERS4*

The coding sequences (CDS) and protein sequences of *ZmERS4* were downloaded from Phytozome.^1^ The molecular weight (Ww) and isoelectric point (pI) were calculated by ExPASy.^2^ The conserved motifs in the *ZmERS4* protein were detected by SMART.^3^ Then, protein sequences between *ZmERS4* and homologous genes were aligned using MEGA v7.0 (a software). A phylogenetic tree was constructed using the neighbor-joining (NJ) method (bootstrap = 1,000; Saitou and Nei, 1987). Gene transcription data of *ZmERS4* at various growth times and in different tissues were downloaded from the Corn database,^4^ which were used to draw a heat map using R software (Paradis et al., 2004).

### Full-Length cDNA Cloning and Vector Construction

*ZmERS4* was cloned by RT-PCR from leaf mRNA from maize leaves by PCR using specific primers (forward: ATGGACGGATGCGATTGC and reverse: TCATACGCTTCTTTGGTACCG) and Primer STAR Mix (Vazyme Biotech Co., Ltd., Nanjing, China). The PCR products were sequenced successfully and constructed into the pCAMBIA 1301 vector to obtain the p35S: *ZmERS4* fusion vector. It was also inserted into the pCAMBIA1305 vector, which included a Cauliflower Mosaic Virus 35S (CaMV35S) promoter to obtain the p35S: *ZmERS4-GFP* fusion vector for subcellular localization. Then, the pCUB-*ZmERS4*-GFP fusion vector and pCUB-GFP vector were constructed, which were transiently expressed in maize protoplasts by homologous recombination for analyzing disease resistance.

### Acquisition of *ZmERS4*-Overexpressing *Arabidopsis*

The pCAMBIA1301 vector carrying 35S: *ZmERS4* was transformed into *Agrobacterium tumefaciens* GV3101, then transgenic *Arabidopsis thaliana* lines were generated by *Agrobacterium*-mediated genetic transformation (Clough and Bent, 1998). T1 seeds were screened on Murashige and Skoog (MS) plates containing 20 μg/ml hygromycin. The DNA of transgenic *A. thaliana* (T1) were extracted by the CTAB method (Clarke, 2009) and used in PCR assays to determine positive transgenic plants. Finally, the homozygous T3 generation derived from transgenic *A. thaliana* was used for subsequent experiments.

### Pathogen Cultivation

*Pseudomonas syringae* pv. tomato DC3000 (*Pst*DC3000) plants were grown at 28°C for 24 h in beef-protein liquid medium. The bacterial suspension (OD_600_ = 0.2) was adjusted using 10 mM MgCl_2_ 6H_2_O, which included 0.02% Silwet L-77.

### Plant Materials and Treatments

Maize lines (B73) were germinated in sand for 2 days at 28°C, and then grown in pots at 25–30°C under a normal photoperiod. Plants at the three-leaf stage were sprayed with 1 mM salicylic acid (SA), 50 μM jasmonic acid (JA), 1 mM ethephon (ET), and the conidial suspension of *Curvularia lunata* (1×10^5^ CFU/ml), and plant leaves at the three-leaf stage were injected with *Pantoea stewartii* suspensions (1×10^6^ CFU/ml). After 0, 3, 6, 12, 24, and 48 h, leaves were harvested and stored at −80°C for RNA extraction. Fifty-four plants were present in each treatment, nine maize plants were harvested and divided into three replicates at different period, and each sample was mixtures of the leaves of the three maize plants. For the tissue-specific expression analysis, maize roots, stems, leaves, leaves, corn silk, and endosperm were obtained and stored at −80°C for RNA extraction. Nine maize plants were harvested and divided into three replicates at different period, and each sample was mixtures of the leaves of the three maize plants.

Wild-type (Col-0) and transgenic *A. thaliana* were sterilized and placed in MS plates at 4°C for 3 days, then transferred to a culture room at 22°C for 10 days. Subsequently, 14-day-old *Arabidopsis* seedlings were transferred to nutritive soil under a 16/8 h light/dark cycle. Then, 4-week-old *A. thaliana* were sprayed with the *Pst*DC3000 suspension; plants treated with 10 mM MgCl_2_ 6H_2_O were used as negative controls. Thirty plants were present in one treatment. Disease severity was recorded 7-day post-infection based on a rating scale of 1–5 (Zha et al., 2019). Among them, 1/2/3/4/5 indicates that the area of sick leaves was 0–20, 21–40, 41–60, 61–80, and more than 80%. The disease index was calculated according to a previously described formula (Ding et al., 2017). Thirty plants in each treatment were divided into three replicates, 10 plants for each replicate. Then plant leaves were harvested and stored at −80°C for RNA extraction at 24 h post-infection (h).

Meanwhile, the bacterium number inside mesophyll tissues was harvested at 2, 4, and 6 days after inoculation and were estimated using the dilution and spread plate method. Nine maize plants were harvested and divided into three replicates at different period, and each sample was mixtures of the leaves of the three maize plants.

To further confirm that *ZmERS4* enhanced plant resistance through the SA signaling pathway, 4-week-old wild and transgenic *A. thaliana* were sprayed with 100 μmol/L 1-aminobenzotriazole (ABT). After 24 h, plants were sprayed with the *Pst*DC3000 suspension; plants pretreated with sterilized distilled water were used as negative controls.

### RNA Extraction and qRT-PCR

RNA was extracted from maize and *Arabidopsis* tissues by the TRIzol method (Thermo Fisher Scientific, United States). RNA quality was evaluated based on the concentrations and electrophoresis strips. In the qRT-PCR assays, each reaction (20 μl total volume) consisted of 2 μl diluted cDNA, 10 μl AceQ qPCR SYBR Green Master Mix (Vazyme Biotech Co., Ltd., Nanjing, China), 1 μl forward and reverse primers each, and 6 μl RNA-free water. Three technical replicates were performed per sample. The qRT-PCR cycling program for validation was as follows: 95°C for 5 min, followed by 40 cycles at 95°C for 10 s, and 60°C for 30 s. Primers were designed using Primer Premier Software v5. 0 (Zha et al., 2019; Supplementary Tables S1 and S2). The relative expression levels of the genes were calculated by the 2^-ΔΔCt^ method, the geometric mean of CT of *Actin* and *GAPDH* was used in calculation (Livak and Schmittgen, 2001) and were displayed using SigmaPlot v10.0 (Livak and Schmittgen, 2001).

### Subcellular Localization Assay of *ZmERS4*

Maize B73 plants were grown in pots at 28°C for 13–14 days in the dark. The third leaf was removed, cut up, and transferred to enzymolysis solution for 6 h. Maize protoplasts were obtained through centrifugation (followed by 100 g for 2 min) and suspended. The p35S: *ZmERS4-GFP* fusion vector and GFP control vector were transformed into protoplasts after 30 min. The reaction was terminated using W5 (Self configuration by the laboratory) solution (154 mmol/L NaCl, 125 mmol/L CaCl_2_, 5 mmol/L KCl, 5 mmol/L glucose, 0.03% MES, pH = 5.8). Then, protoplasts were transferred to round dishes and incubated for 36 h in the dark at room temperature. The fluorescence of protoplasts was observed under a ZEISS LSM 880 Airyscan microscope (Zeiss, Germany).

### Trypan Blue Staining

To detect the extent of damage in ZmERS4-overexpressing *Arabidopsis* leaves treated with the PstDC3000 suspension, trypan blue stain was applied to the leaves. Leaves treated with the PstDC3000 suspension were completely immersed in 2.5 mg/ml trypan blue solution, vacuum-infiltrated for 10 min, and transferred to a water bath at 100°C for 2 min. Then, leaves wrapped in tinfoil were gently placed on a horizontal shaker at 45 rpm for 8–12 h. At the end of the dying process, samples were distained with 2.5 g/ml chloral hydrate solution until colorless (Bhadauria et al., 2010). Finally, distained leaves were affixed for phenotypic observation and photography. Transgenic and wild-type leaves treated with 10 mM MgCl_2_ 6H_2_O or sterilized distilled water were used as negative control.

### *ZmERS4* Overexpression Effects on Resistance Genes in the Maize Protoplast Transient Expression System

After germination, maize plants were grown for 14 days in the dark. Then, the third leaf was removed and cut up for protoplast preparation (Sheen, 2001). Maize protoplasts in which the pCUB-*ZmERS4*-GFP and pCUB-GFP vectors were transiently expressed successfully were subsequently treated with bacterial PAMP flg22 (0.5 μM) for 2 h. The flg22 peptide, QRLSTGSRINSAKDDAAGLQIA (Guan et al., 2013), was synthetized from the Sangon Biotech (Shanghai) Co., Ltd. Gene primers were designed using Primer Premier Software v5.0 (Zha et al., 2019; Supplementary Table S2). RNA of maize protoplasts was extracted to analyze the expression of key genes.

### Detection of SA Content in ZmERS4-Overexpressing *Arabidopsis*

The SA contents of wild and transgenic *A. thaliana* before and at 24 h were monitored by ultra-high-performance liquid chromatography (UPLC), which was performed using an UltiMate3000C_18_ column (100 mm × 2.6 mm, 2.1 μm; Thermo Scientific, United States). SA extraction was performed following previously described methods (Allasia et al., 2018). Samples were detected in the mobile phase [H_2_O (pH = 4): MeOH (20:80)] at a flow rate of 0.2 ml/min and were analyzed at 230 nm. The analysis was performed at the Biotechnology Center of Anhui Agricultural University (China). The total SA content was equal to the sum of free and bound SA. Nine maize plants were harvested and divided into three replicates at different period, and each sample was mixtures of the leaves of the three maize plants.

### Statistical Analysis

Statistical significance of results was calculated using one-way ANOVA followed by least significant difference Duncan’s test at a significant difference level of *p* < 0.05.

## Results

### Identification and Sequence Analysis of *ZmERS4*

**Figure 1:**
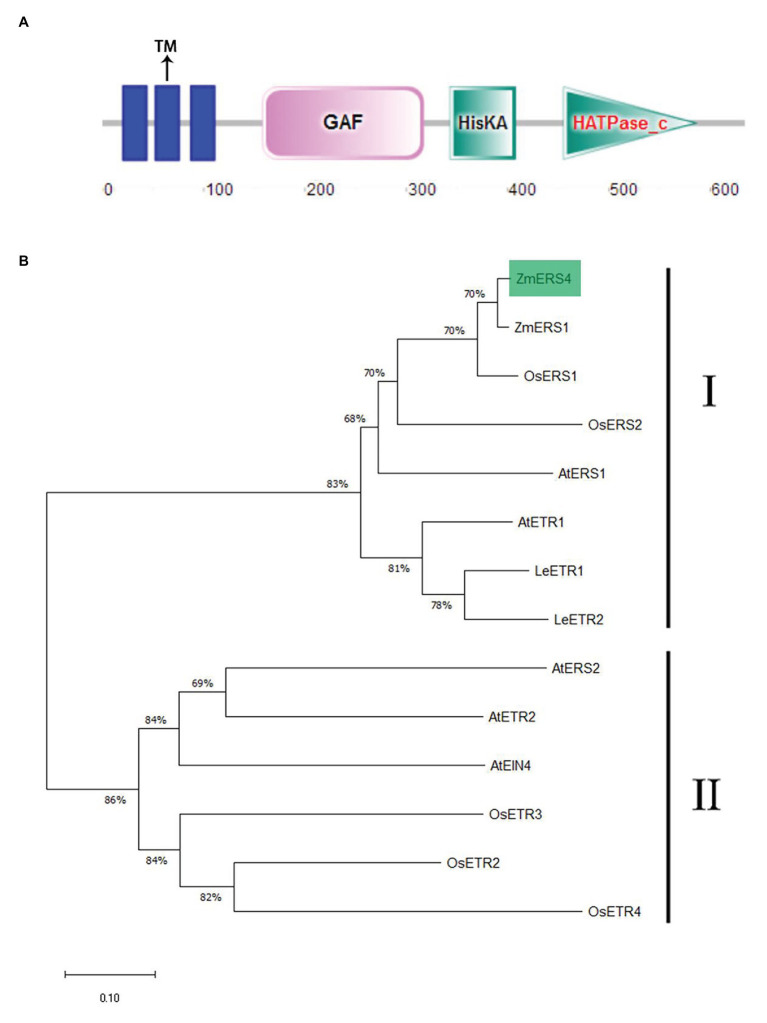
Characterization of ZmERS4. **(A)** The conserved motifs in the ZmERS4 protein were detected by SMART. **(B)** The NJ phylogenetic tree was constructed using ZmERS4 and other ethylene receptor proteins from model species. The phylogenetic tree divided into two clades. ZmERS4 is highlighted in green. Bootstrap values (1,000 replicates) are shown as percentages at the branch nodes. Bar = 0.05. The GenBank accession numbers are as follows: ZmERS4 (NP_001295562.1), ZmERS1 (AY359578) from *Zea mays*, AtERS1 (AT2G40940), AtERS2 (AT1G04310), AtETR1 (AT1G66340), AtETR2 (AT3G23150), and AtEIN4 (AT3G04580) from *Arabidopsis thaliana*, OsERS1 (AY043031.1), OsERS2 (AF460181.1), OsETR2 (AY136816.2), OsETR3 (AY434735.1), and OsETR4 (AY434734.1) from *Oryza sativa*, LeETR1 (U41103), LeETR2(U47279) from *Solanum lycopersicum*.

The full length of the *ZmERS4* (NP_001295562.1) CDS obtained from the Phytozome database was 1905 bp in length, encoded 634 amino acids, and was located on chromosome 5. The pI and Ww of the *ZmERS4* protein were predicted to be 7.07 and 70.5 3 kDa, respectively. It contained a typical conserved domain of an ethylene receptor protein, including three transmembrane domains, GAF, and HisKA (Figure 1A; Supplementary Figure S1), showing similarity to bacterial two-component His kinases (Chang et al., 1993; Sakai et al., 1998). The amino acid sequences of *ZmERS4* were compared to the ethylene receptor protein families of *A. thaliana*, *Oryza sativa*, *Z. mays*, and *Solanum lycopersicum*. According to the phylogenetic relationships and bootstrap values, the ethylene receptor proteins in the four surveyed species divided into two subfamilies. Furthermore, *ZmERS4* belonged to the first subfamily of ethylene receptor proteins and was highly homologous with ZmERS1 from *Z. mays* (Figure 1B).

### Expression of *ZmERS4* in Different Maize Tissues

**Figure 2:**
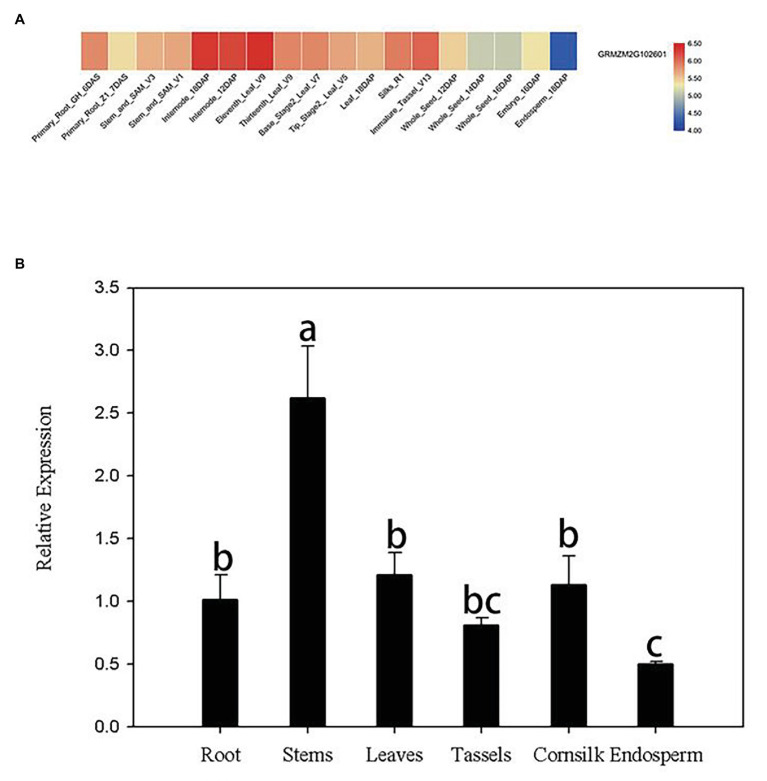
Tissue-specific expression patterns of ZmERS4. **(A)** Heat map of ZmERS4 gene expression in maize. High, medium, and low expression levels are indicated in red, gray, and blue, respectively. GH, greenhouse; V, vegetative growth stage; R, reproductive growth stage; DAS, days after sowing; DAP, days after pollination. **(B)** Spatial expression patterns of ZmERS4 in maize. Data are presented as the mean ± SD.

Publicly available microarray data of maize were used to detect tissue-specific expression patterns of *ZmERS4*. Data from the transcriptome database indicated that *ZmERS4* was expressed in different tissues at distinct stages and the expression levels were higher in the leaves, corn silks, and stems (Figure 2A). Furthermore, the tissue-specific expression profiles were verified by qRT-PCR using six tissues from the inbred maize B73 line. Results revealed that the expression levels of *ZmERS4* in the six tissues were consistent with the chip data and *ZmERS4* was expressed at higher levels in the stems, leaves, and corn silks (Figure 2B). These results suggested that *ZmERS4* was expressed in all corn growth stages.

### *ZmERS4* Expression Was Induced by Different Hormones and Pathogenic Microorganism

**Figure 3:**
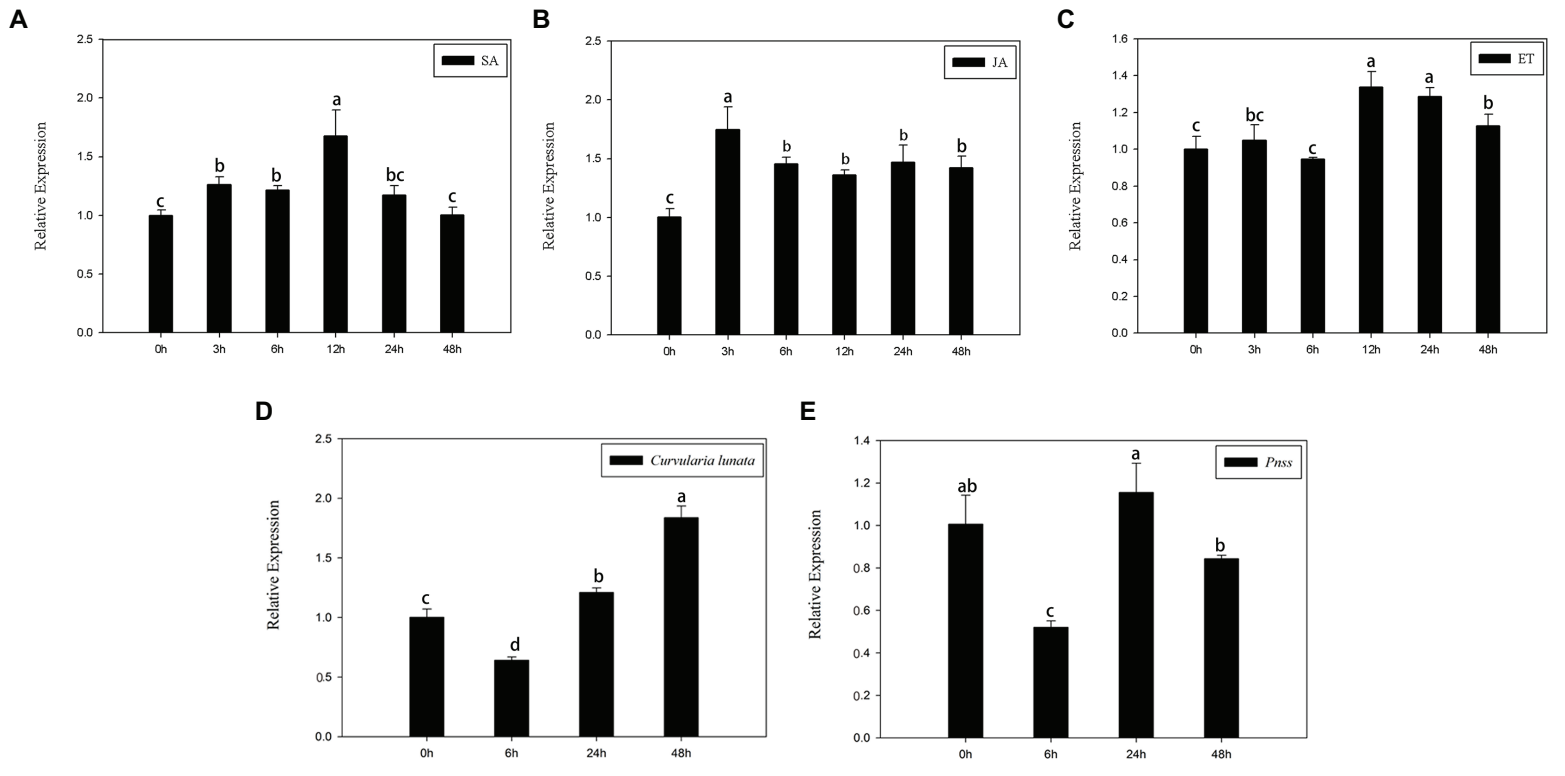
Relative expression levels of ZmERS4 under three different treatments at six time points. **(A)** Salicylic acid (SA) spraying, **(B)** jasmonic acid (JA) spraying, **(C)** ethephon (ET) spraying, **(D)** The conidial suspension of *Curvularia lunata* (1 × 10^5^ CFU/ml) spraying, and **(E)** *Pantoea stewartii* suspensions (1 × 10^6^ CFU/ml). ZmActin1 and GADPH were used as reference genes for normalization. The expression levels at 0 h were used as the control and assigned a value of 1. Data are presented as the mean ± SD. Three biological replicates were performed.

SA, JA, and ethylene, three typical stress hormones that mediate plant defenses in response to biotic stress, were applied to determine whether *ZmERS4* is involved in pathogen resistance. At the three-leaf stage, the transcript levels of *ZmERS4* gradually increased 3–12 h after SA application and its expression was increased by 1.70-fold in SA-treated plants after 12 h compared to the pre-treatment 0 h control group (Figure 3A). *ZmERS4* gradually increased 0–3 h after JA inoculation and its transcripts were increased by 1.75-fold 3 h after JA application. Specifically, *ZmERS4* transcripts were increased by 1.46-, 1.37-, 1.47-, and 1.43-fold after 6, 12, 24, and 48 h in JA-treated plants, respectively (Figure 3B). Moreover, the expression of *ZmERS4* in ET-treated plants after 3 and 6 h were not significantly different when compared to 0 h, and the expression of *ZmERS4* was 1.29-fold higher at 12 h after ET application, and then slowly declined (Figure 3C). It seemed that there was an early response of *ZmERS4* to JA (3 h), later to SA (12 h) and after to ET (12–48 h), the results indicated that *ZmERS4* might respond to all three hormones with different timing of pattern and expression.

Meanwhile, the transcript levels of *ZmERS4* gradually increased 6–48 h after *C. lunata* application (Figure 3D), and the expression of *ZmERS4* in *C. lunata*-treated after 48 h were 1.84-fold higher compared to 0 h. However, the expression of *ZmERS4* in *P. stewartii*-treated plants were 1.16-fold higher at 24 h (Figure 3E). These results showed that *ZmERS4* may participate in plant disease resistance, but the underlying mechanisms require further investigation.

### Subcellular Localization Analysis of *ZmERS4*

**Figure 4:**
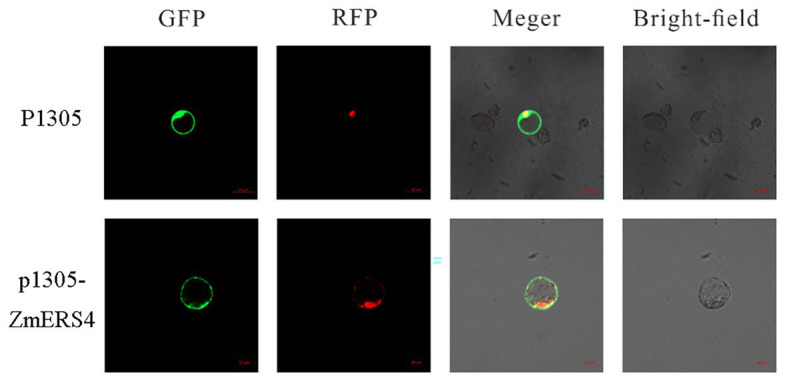
Subcellular localization of the ZmERS4-GFP fusion protein in maize protoplasts. GFP: green fluorescent protein; RFP: nuclear marker pSAT6-mCherry-VirD2NLS. Scale bar = 20 μm.

To verify the location of *ZmERS4* in cells, the p1305-*ZmERS4*-GFP fusion vector was constructed, in which the *ZmERS4* CDS without the termination codon was fused upstream of the GFP reporter under control of the CaMV35S promoter (Supplementary Figure S2). The recombinant plasmid was introduced into maize protoplasts with a nuclear localization plasmid, and then observed under a laser confocal microscope. The green fluorescent signal of *ZmERS4*-GFP was observed around the plasma membrane without overlapping the nuclear marker. Because the cytoplasm can be “pushed” against the plasma membrane in vacuolated cells, the ZmERS4 protein was localized in plasma membrane or cytoplasm (Figure 4).

### *ZmERS4* Conferred Increased Resistance to *Pst*DC3000 in *Arabidopsis*

**Figure 5:**
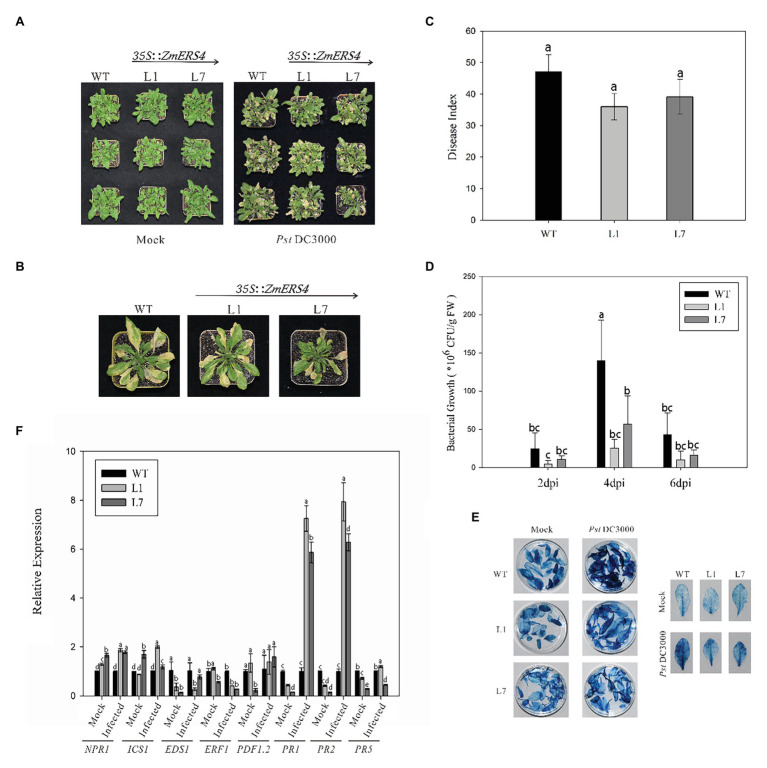
ZmERS4 transgenic *Arabidopsis* plants conferred enhanced resistance to PstDC3000. **(A,B)** Phenotypes of ZmERS4 transgenic and wild-type *Arabidopsis* at 7 days. **(C)** The disease indexes of different groups. **(D)** The population density of bacterium in *Arabidopsis* leaves of different groups at 2, 4, and 6 days. Values of *p* were determined by a two-tailed Student’s *t* test assuming equal variances (*p < 0.05); three biological replicates were performed. **(E)** Trypan blue staining in *Arabidopsis* leaves of different groups at 7 days. **(F)** Expression levels of marker genes in SA, JA, and ET-mediated signaling pathways at 24 h. AtActin2 and AtTUB4 were used as reference genes for normalization. The expression levels in wild-type plants were used as the control and assigned a value of 1. Data are presented as the mean ± SD based on three biological replicates.

To further investigate the function of *ZmERS4*, the p1301-*ZmERS4* fusion expression vector was constructed (Supplementary Figure S3). *Agrobacterium tumefaciens* cells containing 35S: *ZmERS4* were transformed into wild-type *Arabidopsis* (Supplementary Figure S4). Two independent T3 homozygous lines (L1 and L7) were selected for *ZmERS4*. Under normal growth conditions, L1 and L7 exhibited similar growth behavior to the background wild-type control, Col-0 (Figure 5A). *Pst*DC3000 suspension was sprayed on the leaves of L1 and L7 (2 ml per plant). Symptoms appeared in all groups at 3 days. At 7 days, small tan necrotic spots on the leaves were observed. Compared to the negative control (Figure 5A), the L1 and L7 had less spots (Figures 5A,B). The plant disease severity assessment was also conducted using the disease index 7 days with *Pst*DC3000. The disease indexes of L1 and L7 were 35.94 and 39.09, respectively. L1 had a significantly lower disease index (47.17) compared to wild-type *Arabidopsis* (Figure 5C). These results indicated that the overexpressing lines conferred enhanced resistance compared to wild-type *Arabidopsis*.

To determine the relationship between the number of bacteria in *Arabidopsis thaliana* overexpressing *ZmERS4* and plant disease resistance, the dilution plating method was used and the population density of bacterium in *Arabidopsis* leaves was detected. The population densities in L1 and L7 were consistently lower than wild-type *Arabidopsis* at 2, 4, and 6 days, respectively. The population density of L1 reached 2.52 × 10^7^ CFU/g in leaf tissues at 4 days and remained at 1.37 × 10^7^ CFU/g until 6 days compared to 1.39 × 10^8^ CFU/g at 4 days and 3.71 × 10^7^ CFU/g at 6 days in wild-type *Arabidopsis*, respectively (Figure 5D). These results showed that *ZmERS4*, when over-expressed in *A. thaliana*, partially increases resistance to PstDC3000.

Plant hypersensitive cell death is an important event in early plant pathogen invasion. Infection by site and adjacent site of programed cell death to restrict pathogen expansion causes plants to develop local acquired resistance (LAR) and SAR and plays an important role in plant resistance to pathogen invasion. After staining WT plant and transgenic *A. thaliana* lines L1 and L7 inoculated with *Pst*DC3000 using trypan blue, findings showed that the transgenic *A. thaliana* lines and wild-type plants exhibited necrotic cell accumulation and appeared dark blue (Figure 5E). However, after *Pst*DC3000 treatment, wild-type plants inoculated with *Pst*DC3000 were dark blue compared to the light blue leaves of the transgenic lines (Figure 5E). According to the disease indexes of different treatment groups (Figure 5C), the leaf damage degree of transgenic plants was lower than wild-type plants and the trypan blue staining results confirmed these findings. These results suggest that HR was induced in heterologous expression of *ZmERS4*- in *A. thaliana* in response to *Pst*DC3000.

To investigate the signaling pathways in which *ZmERS4* was involved in and conferred enhanced resistance in transgenic *Arabidopsis*, the expression levels of target plant genes that are known to function in the SA, JA, or ET pathways were analyzed at 24 h after (Dinolfo et al., 2017; Zheng et al., 2019).when the control groups were treated with 10 mM MgCl_2_ 6H_2_O, the *EDS1*, *PR1*, *PR2*, and *PR5* were expressed at lower levels in L1 and L7 than wild-type plants, the *NPR1* were expressed at higher levels in L1 and L7 than wild-type plants (Figure 5F). However, after the control groups were infected with *Pst*DC3000, the *PR1* was strongly induced in L1 and L7, and was 7.26- and 5.87-fold higher compared to wild-type plants, respectively (Figure 5F). *PR2* expression in L1 and L7 were similar to *PR1* and was induced 7.93-fold and 6.28-fold higher, respectively (Figure 5F). Moreover, *NPR1*, *ICS1*, and *PDF1.2* were slightly induced in L1 and L7 at 24 h (Figure 5F). *EDS1* and *PR5* transcripts did not exhibit apparent changes before and after inoculation (Figure 5F). And the expression levels of *Ethylene Response Factor1* (*ERF1*) in L1 and L7 were lower than the negative controls (Figure 5F). These results showed that *PR1* and *PR2* were concurrently highly expressed in *ZmERS4*-overexpressing lines after pathogen inoculation, suggesting that *ZmERS4* may be involved in plant defense mechanisms by activating SA-dependent signaling pathways.

**Figure 6:**
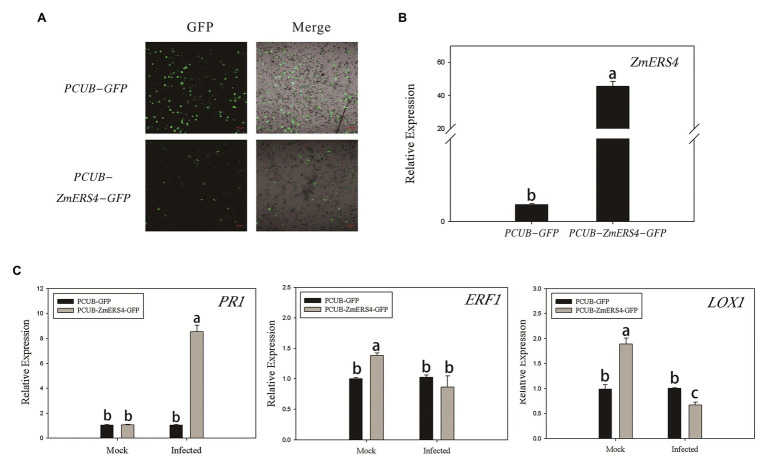
The transient overexpression of ZmERS4 in maize protoplasts was involved in plant defense responses through the SA signaling pathway. **(A)** The pCUB-ZmERS4-GFP and pCUB-GFP vectors were transiently expressed in maize protoplasts with a transformation efficiency >50%. GFP, green fluorescent protein. Scale bar = 100 μm. **(B)** Expression levels of ZmERS4 extracted from maize protoplasts that did and did not overexpress ZmERS4. **(C)** Expression levels of the marker genes in the three signaling pathways induced by 0.5 μM flg22 after 2 h. ZmActin and ZmGADPH were used as reference genes for normalization. The expression levels of the protoplasts that expressed pCUB-GFP were used as the control and were assigned a value of 1. The data are presented as the mean ± SD based on three biological replicates.

The pCUB-*ZmERS4*-GFP and pCUB-GFP (control) vectors were constructed and transiently expressed in maize protoplasts (Figure 6A). The expression levels of *ZmERS4* increased by 50-fold in maize protoplasts that transiently expressed the pCUB-*ZmERS4*-GFP vector compared to the control (Figure 6B). The expression of *PR1* (a SA-responsive marker gene), lipoxygenase 1(*LOX1*; a JA-responsive marker gene) and *ERF1*, which are expressed in plant defense mechanisms (Erb et al., 2009; Xie et al., 2019), were used in this study. As demonstrated by qRT-PCR, the expression of *PR1* was not significantly different between protoplasts that transiently expressed pCUB-*ZmERS4*-GFP or empty vectors without pathogen inoculation, while the expression of *ERF1* and *LOX1* in protoplasts that expressed pCUB-*ZmERS4*-GFP was slightly higher than protoplasts that expressed empty vectors. However, the expression of *PR1* in pCUB-*ZmERS4*-GFP protoplasts treated with 0.5 μM flg22 peptide, an elicitor of *P. syringae* (Guan et al., 2013), was strongly induced 8-fold higher compared to the pCUB-GFP vector (Figure 6C). Additionally, the expression of *ERF1* and *LOX1* in pCUB- *ZmERS4*-GFP protoplasts treated with 0.5 μM flg22 peptide was lower than the pCUB-GFP vector (Figure 6C). These results further corroborated that *ZmERS4* participates in plant defense responses through the SA signaling pathway.

### *ZmERS4*-Overexpressing *Arabidopsis* Exhibited Increased SA Content

**Figure 7:**
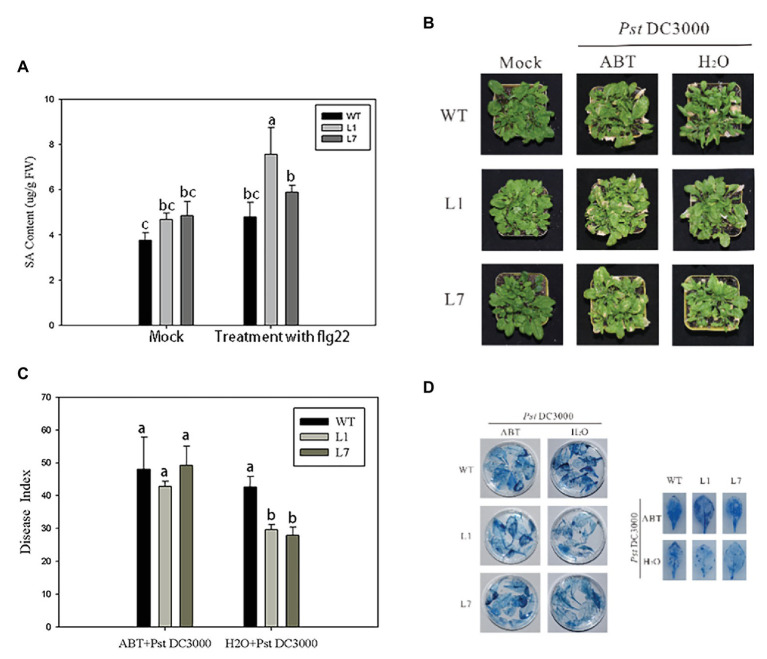
ZmERS4 transgenic *Arabidopsis thaliana* exhibited improved resistance to PstDC3000 by the SA-mediated signaling pathway. **(A)** SA contents were extracted from wild-type and ZmERS4 transgenic *Arabidopsis* at 24 h. **(B)** Phenotypes of wild-type and ZmERS4 transgenic *Arabidopsis* at 7 days; plants were pretreated with ABT for 24 h. **(C)** Disease indexes of the different groups shown in **(B)**. **(D)** Trypan blue staining of the *Arabidopsis* leaves in the different groups shown in **(B)**.

The preliminary results showed that *ZmERS4* played an important role in plant defense responses through the SA signaling pathway; however, changes in the SA contents of transgenic plants after *Pst*DC3000 infection were unclear. Thus, UPLC was used to detect the SA contents in *ZmERS4*-overexpressing *Arabidopsis*. The absorption peaks of the SA extracts from L1, L7, and wild-type *Arabidopsis* appeared at ~1.43 s (Supplementary Figure S5), which was consistent with the peak time of the standard SA. The total SA contents in L1 and L7 were generally higher than wild-type plants, which were treated with and without *Pst*DC3000. Findings showed the SA contents in L1 and L7, which were 4.512 and 4.901 μg/g FW, were higher than that of the wild-type plants (3.848 μg/g FW) without pathogen infection. However, when the plants were infected by *Pst*DC3000, the SA contents in L1 rapidly accumulated at 24 h (Figure 7A). The total SA contents in L1 and L7 reached 7.826 and 5.640 μg/g FW at 24 h compared to wild-type plants (4.54 μg/g FW) inoculated with the pathogen, respectively (Figure 7A). These results indicated that the resistance to *Pst*DC3000 in L1 and L7 was enhanced due to SA accumulation.

### Effects of ABT on Pathogen Resistance in *ZmERS4*-Overexpressing *Arabidopsis*

ABT is an inhibitor of SA biosynthesis and can effectively control SA biosynthesis in plants (Wang et al., 2019). To confirm that *ZmERS4* enhanced *A. thaliana* resistance to *Pst*DC3000 through the SA signaling pathway, *ZmERS4*-overexpressing lines and wild-type plants were pretreated with 100 μmol/L ABT for 24 h. Then, plants were sprayed with the *Pst*DC3000 suspension. The disease indexes were estimated at 7 days. As expected, both overexpressing and wild-type plants pretreated with ABT exhibited more severe symptoms (Figure 7B). The disease indexes of L1, L7, and wild-type *Arabidopsis* were 42.76, 49.26, and 48.06, respectively (Figure 7C), while the disease indexes of the lines pretreated with sterilized distilled water were 29.65, 27.87, and 42.67, respectively (Figure 7C). Dead cells in the leaves of plants pretreated with ABT and sterilized distilled water were further detected by trypan blue staining. Compared to the negative controls, the dead cells in the treatment groups pretreated with ABT were dark blue due to a higher number of larger areas of these spots (Figure 7D). Clearly, ABT application reduced plant resistance to pathogen infection, further indicating that the overexpression of *ZmERS4* in *Arabidopsis* enhanced resistance to *Pst*DC3000 by activating the SA signaling pathway.

## Discussion

A candidate disease-resistant gene, *ZmERS4*, was explored in our previous study. According to the protein domain prediction and sequence homology analysis, our results showed that *ZmERS4* could encode a protein similar to ethylene receptor, showing the highest sequence similarity with OsERS1 in rice. Ethylene receptor genes have unique expression patterns in different plant tissues. Previous studies reported that ETR1 and ERS1, members of the ethylene receptor ETR1 subfamily, are expressed ubiquitously in *Arabidopsis* plants. The expression of AtETR1 is constitutive and ethylene independent, while AtERS1 is tissue specific, ethylene dependent, and developmentally regulated, which was previously observed in the leaves, stems, floral organs, and especially the procambium cells, and very strong expression was observed in the locules of the anthers (Hua et al., 1998). Our results demonstrated that *ZmERS4* was expressed in maize tissues and predominantly expressed in the stems and leaves, suggesting that *ZmERS4* was expressed in all stages of maize growth and development. The results were consistent with the findings of ETR1 and ERS1.

Ethylene receptor genes play important roles in plant responses to hormones. Different types of ethylene receptor genes exhibit different expression patterns in response to hormones. It was reported that ETR- and ERS-type genes, *PpETR1* and *PpERS1*, of peach fruit showed different expression patterns in response to ethylene and propylene, such that propylene treatment did not affect the transcript levels of *PpETR1*, while *PpERS1* was upregulated in the pit hardening and climacteric ripening stages (Rasori et al., 2002). Ethylene receptor proteins, which perceive ethylene signals, are located upstream of signal transduction and play negative regulatory roles in the transmission of ethylene signals (Hua et al., 1998). In this study, the expression of *ZmERS4* was induced after SA or JA application. However, as an ethylene receptor protein, its transcript levels did not change after ET treatment, suggesting that ET did not affect the transcript levels of *ZmERS4*, which was consistent with the findings of previous studies.

To date, research on plant ethylene receptor proteins has mainly focused on the regulation of growth and development, changing the flowering period, and delaying senescence and death (Gallie, 2015; Chen et al., 2020). A few reports are known about the roles of ethylene receptor genes in plant disease resistance. Here, we identified some characteristic genes using *ZmERS4*-overexpressing *Arabidopsis* inoculated with *Pst*DC3000, which are similar to known or deduced functions involved in SA, JA, and ET pathways (Niu et al., 2011). These genes are also suspected to play roles in generating signals for the activation of certain defense responses and protecting plants from damage associated with these defense responses. We found that the defense-related genes, *PR1* and *PR2*, were highly expressed in the leaves of *ZmERS4*-overexpressing *Arabidopsis*, while the expression of other genes did not increase. *PR1* and *PR2* are marker genes of the SA-mediated signaling pathway, and SA can activate the expression of PR genes. Thus, we hypothesized that *ZmERS4*-overexpressing *Arabidopsis* confer enhanced pathogen resistance by activating an induced response through the SA signaling pathway. Interestingly, the SA contents in *ZmERS4*-overexpressing plants rapidly accumulated at 24 h and the improved resistance of *ZmERS4*-overexpressing *Arabidopsis* was repressed by the application of 100 μmol/L ABT, a SA biosynthetic inhibitor. Moreover, the expression of *PR1* in pCUB-*ZmERS4*-GFP protoplasts treated with 0.5 μM flg22 peptide was strongly induced expression by 8-fold higher. These results showed that the overexpression of *ZmERS4* elevated SA levels after pathogen infection and increased the expression of PR genes, suggesting that *ZmERS4* was involved in plant defense processes through the SA signaling pathway and was subjected to SA feedback regulation.

According to a previous study, SA affects plant development and growth, and activates HR to promote cell death, resulting in pathogen resistance (Vlot et al., 2009; Kazan and Lyons, 2014; Han and Kahmann, 2019). SA also plays a role in abiotic stress responses (Alonso-Ramírez et al., 2009). Previous studies reported the SA was associated with ethylene signaling (Devadas et al., 2002; Yin et al., 2013). For example, SA and ethylene coordinate plant pathogen responses (Devadas et al., 2002), and many ethylene signaling components, such as tomato Pti4 (Gu et al., 2000) and apple MdETR1, MdERS1, and MdCTR1 (Li et al., 2006), are transcriptionally regulated after SA treatment. Yin et al. investigated the effects of acetylsalicylic acid (ASA) on kiwifruit ethylene biosynthesis and signaling, and found that the *ERF*s, *AdERF1*, *AdERF3*, and *AdERF12*, were characterized as ASA-responsive genes, suggesting that the effect of SA on fruit ripening may involve the regulation of genes associated with ethylene signaling (Yin et al., 2013). The results of this study indicated that *ZmERS4* overexpressing plants conferred enhanced disease resistance to *Pst*DC3000 by promoting SA accumulation, thus supporting previous findings (Devadas et al., 2002; Yin et al., 2013).

However, information regarding the molecular mechanisms of *ZmERS4* overexpression in the modulation of SA signaling components in plants remains lacking. It was reported that the ethylene receptor protein, OsERS1, in *O. sativa* may interact with the rice protein, OsTPR1, and this interaction plays a key role in rice ethylene signaling, as well as influences rice growth and development (Yi, 2013). The *Arabidopsis* protein, AtTRP1, interacts preferentially with the *Arabidopsis* ethylene receptor, ERS1, and the overexpression of AtTRP1 enhances some ethylene responses (Lin et al., 2009). Therefore, we speculate that *ZmERS4* may interact with certain factors or proteins to respond to ethylene signals. However, this notion requires further investigation in order to elucidate the mechanisms of ethylene signal responses in the *ZmERS4* gene.

## Data Availability Statement

The original contributions presented in the study are included in the article/Supplementary Material, further inquiries can be directed to the corresponding author.

## Author Contributions

TH has made major contributions to the conception and design of the study. TH and XL contributed to the acquisition, analysis, or interpretation of the data. TH, XL, SX, CH, HJ, and TD performed the experiments and participated to the interpretation of data. TH, XL, and TD wrote the manuscript. All authors read and approved the final manuscript.

### Conflict of Interest

The authors declare that the research was conducted in the absence of any commercial or financial relationships that could be construed as a potential conflict of interest.

## Footnotes

^1^http://phytozome.jgi.doe.gov/pz/portal.html

^2^https://web.expasy.org/compute_pi/

^3^http://smart.embl-heidelberg.de/

^4^https://www.maizegdb.org/
