# Supplementary material for: Isolation of the ZmERS4 Gene From Maize and Its Functional Analysis in Transgenic Plants
Source: Front Microbiol. 2021 Mar 12;12:632908. doi: 10.3389/fmicb.2021.632908 (PMC7994261; doi:10.3389/fmicb.2021.632908)
Supplement: Supplementary file 1 [file Data_Sheet_1.pdf]

Supplementary Materials

Figure S1. Molecular characterization of *ZmERS4*.

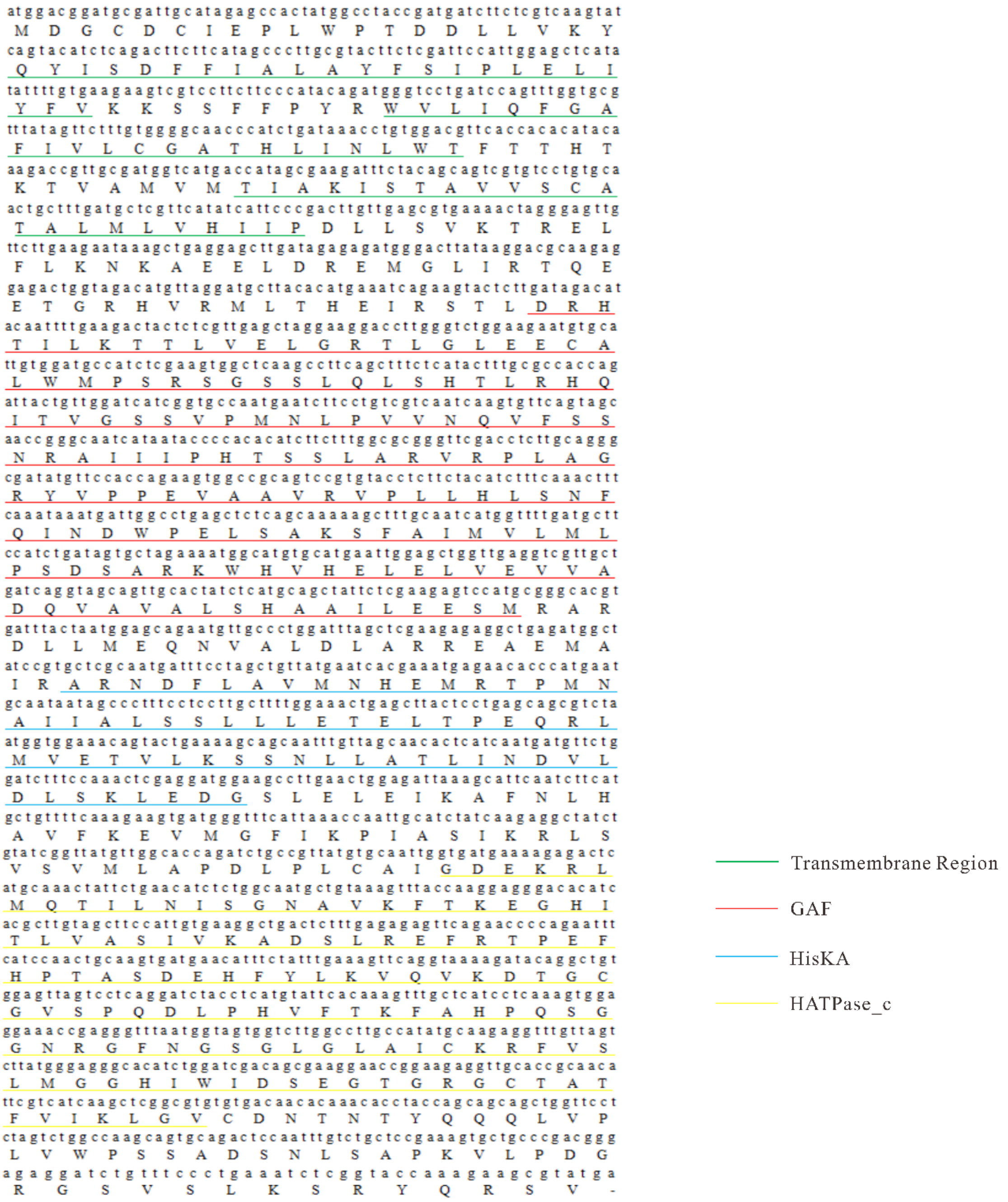

Figure S2. Construction of the p1305-*ZmERS44*-GFP vector. (A) The pCAMBIA1305 vector included a CaMV35S promoter and GFP tag; *ZmERS4* was inserted in front of the GFP. (B) Electrophoretic map of enzyme digestion validation. M: 5K DNA marker; 1: Destination strip.

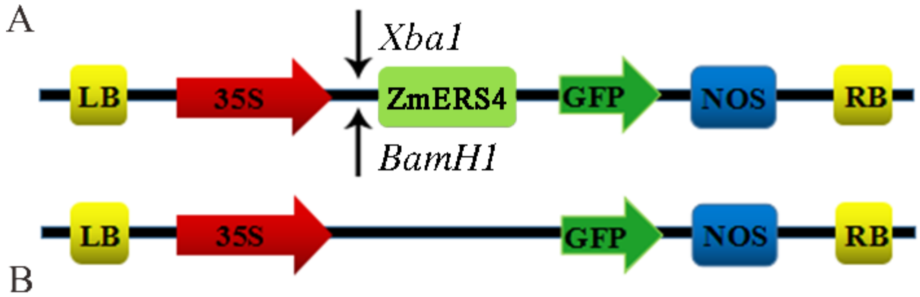

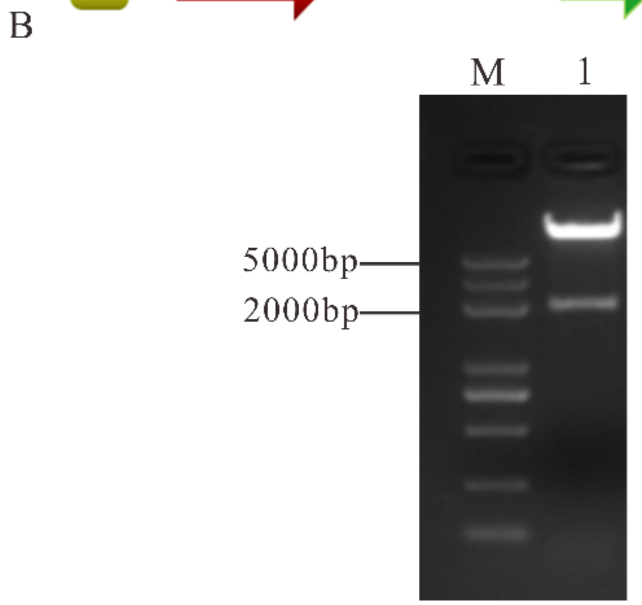

Figure S3. Construction of the p1301-*ZmERS4* vector. (A) The pCAMBIA1301 vector included a CaMV35S promoter, and Hyg and GUS tags. (B) Electrophoretic map of enzyme digestion validation. M: 5K DNA marker; 1: Destination strip.

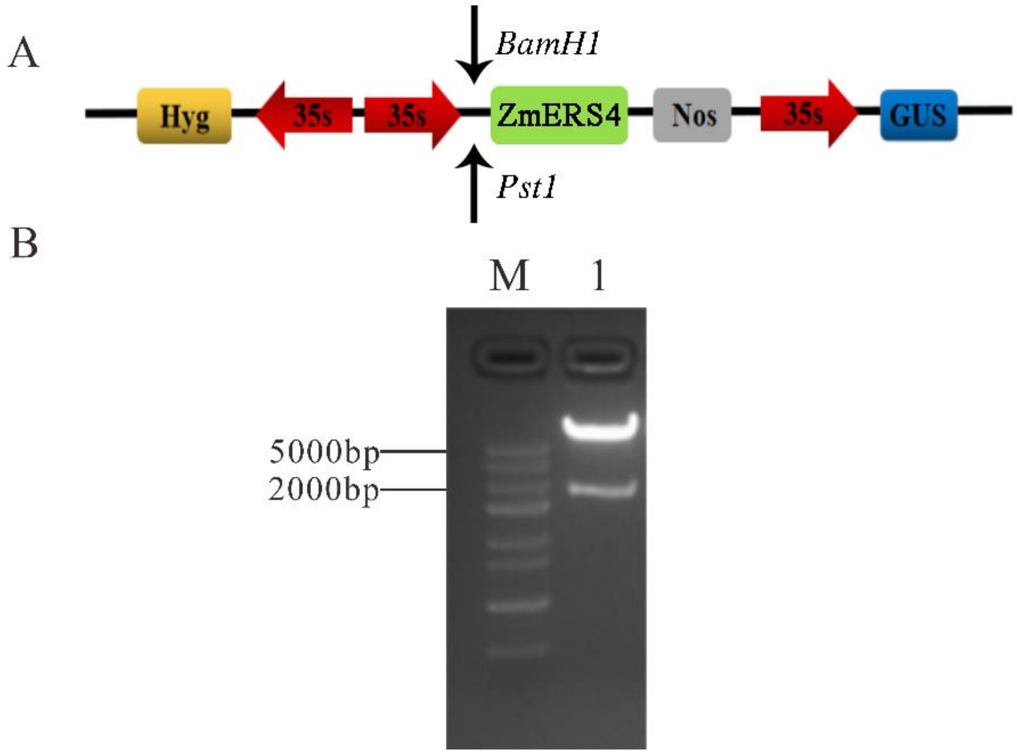

Figure S4. Detection of *ZmERS4* transgenic *A. thaliana*. (A) GUS histochemical staining. WT: Negative control; 1301a: Positive control. (B) PCR analysis of *ZmERS4*. (C) PCR analysis of *GUS*. M: 5K DNA marker; +: Positive control; -: Negative control.

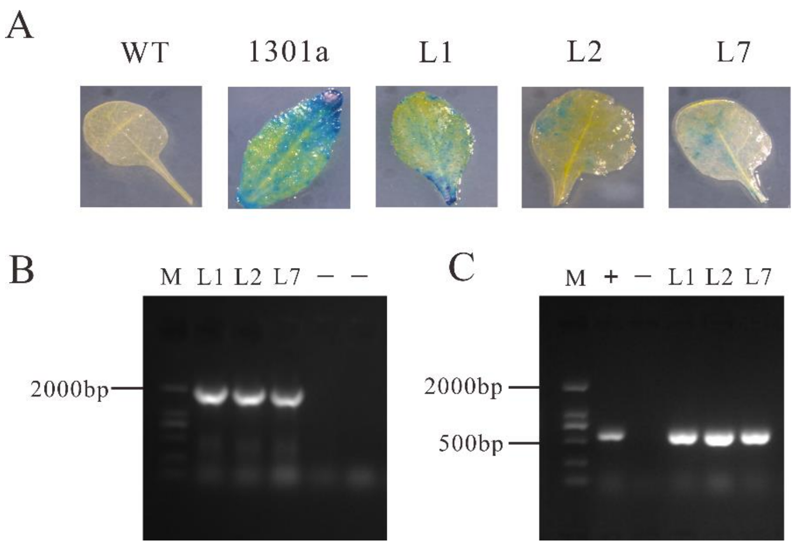

Figure S5. Chromatogram of SA extracted from *A. thaliana*. (A) SA chromatogram of standard SA. (B) SA chromatogram of Wild-type (Col-0) and transgenic *A. thaliana* L1 and L7. (C) The SA chromatogram of Wild-type (Col-0) and transgenic *A. thaliana* L1 and L7 infected with *pst*DC3000. arrow indicates the SA peak; mAU indicates the peak height.

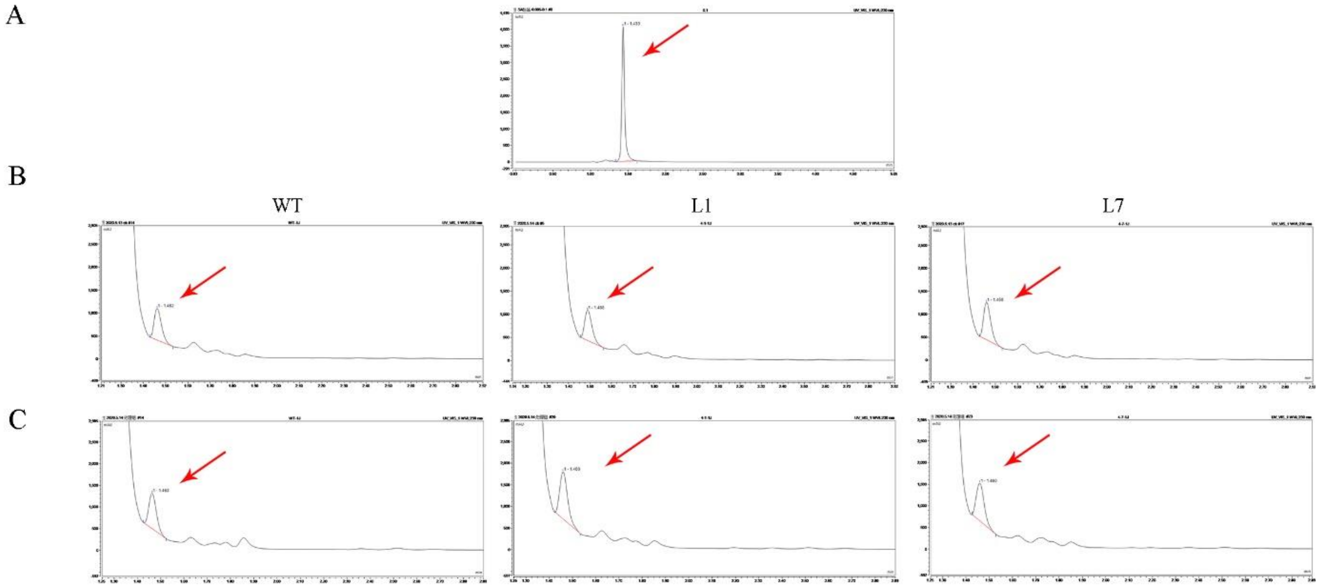

Table S1. Primer sequences used in the qRT-PCR analysis of *A. thaliana*.

Gene name	Forward primer (5’-3’)	Reverse primer (5’-3’)
ICS1	GCTTGGCTAGCACAGTTACAGC	CACTGCAGACACCTAATTGAGTCC
EDS1	GCTCAATGACCTTGGAGTGAGC	TCTTCCTCTAATGCAGCTTGAACG
NPR1	AACGATTCTTCCCGCGCTGTTC	TTCTCCGCAAGCCAGTTGAGTC
PDF1.2	CTTGTTCTCTTTGCTGCTTTCGAC	TTGGCTCCTTCAAGGTTAATGCAC
ERF1	CCTTCCGAT CAA ATC CGT AAG	TCCCGAGCC AAA CCC TAA TAC
PR1	ACACGTGCAATGGAGTTTGT	TGCAACTGATTATGGTTCCA
PR2	ATGTGGGTTAGCGAGAAGGC	TTGGCACATCCGAGTCTCAC
PR5	GAAACTTGTCCTCCCACGG	CTCGTTTCGTCGTCATAAGC
AtActin2	GGTAACATTGTGCTCAGTGGTGG	AACGACCTTAATCTTCATGCTTGC
AtTUB4-F	CGAAAACGCTGACGAGTGTA	CCTTGGGAATGGGATAAGGT

Table S2. Primer sequences used in the qRT-PCR analysis of *ZmERS4* and maize protoplasts.

Gene name	Gene ID	Forward primer (5’-3’)	Reverse primer (5’-3’)
ZmERS4	*NP_001295562.1*	TTGTTAGTCTTATGGGAGGGC	TGCTGGTAGGTGTTTGTGTTGTC
PR1	—	CTGGGTGTCCGAGAAGCAGT	CGGGTTGTAGCTGCAGATGAT
ERF1	*Zm00001d035835*	GGTCAATTTCCGGGACGAAGC	CGTTGCCACAAGCAGTTGGAGTAG
LOX1	*Zm00001d042541*	GCGACACCATGACCATCAAC	GCTCGGTGAAGTTCCAGCTC
GADPH	—	CTTCGGCATTGTTGAGGGTTTG	TCCTTGGCTGAGGGTCCGTC
ZmActin1	—	GGATTGCCGATCGTATGAG	GAGCCACCGATCCAGACACT
